# Use of transcutaneous capnography in critically ILL patients

**DOI:** 10.1186/2197-425X-3-S1-A389

**Published:** 2015-10-01

**Authors:** G Morales Varas, H Marin Mateos, S Chancón Alves, M Castillo Jaramillo, JA Sánchez-Izquierdo Riera, JC Montejo González

**Affiliations:** Hospital 12 de Octubre, Madrid, Spain

## Introduction

Assess alveolar ventilation is a routine when patients are mechanically ventilated. Transdermal devices that measure CO_2_ pressure (PtcCO_2_) are can be used but should be evaluated in critically ill patients with altered tissue perfusion.

## Objectives

To determine the usefulness of transcutaneous capnography in patients with mechanical ventilation.

## Methods

A prospective-observational, single-center study was carried out in the medical-surgical intensive care unit (ICU) at university hospital. Population: All patients over 16 years old, who required respiratory support with invasive mechanical ventilation, the measurement of PtcC02 is done by SenTec Digital Monitor and their results were compared with samples of blood gas analysis (PaC02) in patients with different hemodynamic conditions. Study period: June 2014 to December 2014. Demographic and clinical date included reason for admission, body temperature, requirement of vasoactive drugs, neuromuscular blockers and maneuvers using for treat to the refractory hypoxemia such as prone position ventilation and veno-venous extracorporeal membrane oxygenation (ECMOv-v). Statistical analysis of the results was performed using SPSS software version 22. Quantitative variables were expressed as mean and SD. Qualitative variables were expressed as percentages. Agreement between PaC02 and PtcC02 was evaluated using lineal regression analysis.

## Results

A total of 78 samples were analyzed. Mean age was 58 (SD 9.9) years, with an average stay in ICU of 24.8 (SD 13.3) days, the main reason for admission was respiratory distress syndrome (ARDS) with 42.3% of the patients, 24.3% of them were ventilated in prone position and 48.4% received support with ECMO v-v. Mean of PtcC02 vs PaC02 was 54.2 (SD 11.2) mmHg / 55.4 (SD 12.8) mmHg respectively. PtcC02 was highly correlated with PaC02 (r = 0.79; p < 0.001), as determined to by lineal regression analysis. 37.2% of the samples under support with vasoactive drugs did not affect PtcC02 accuracy (r = 0.75; p < 0.001) relative to the PaC02.

## Conclusions

In our study, continuous monitoring of the trends of PtcC02 constitutes an useful method for assessing alveolar ventilation in critically ill mechanically ventilated even in situations of hemodynamic inestability.
